# Edited HSPCs rescue frataxin deficiency and inflammation-driven multisystemic pathology in Friedreich’s ataxia mice

**DOI:** 10.1172/JCI209546

**Published:** 2026-10-15

**Authors:** Anusha Sivakumar, Alexis N. Corl, Keneth Jordan Muñoz, Subhashini Arumugam, Betul B. Gunes, Angelyn B. Solis, Rishika Murthy, Lilas Rony El-Hachem, Donald P. Pizzo, Ian A. Blair, Teerapat Rojsajjakul, Clementina Mesaros, Xinlong Wan, Joseph N. Rainaldi, Rafael A. Badell-Grau, Veenita Khare, Kevin Eric Thai, Kavya Biederman, Stephanie Cherqui

**Affiliations:** 1 Department of Pediatrics, Division of Genetics, and; 2 Department of Pathology, University of California, San Diego, La Jolla, California, USA.; 3 Penn/CHOP, Friedreich’s Ataxia Center of Excellence, Philadelphia, Pennsylvania, USA.; 4 Center of Excellence in Environmental Toxicology, Department of Systems Pharmacology and Translational Therapeutics, Perelman School of Medicine, University of Pennsylvania, Philadelphia, Pennsylvania, USA.

**Keywords:** Cell biology, Inflammation, Neuroscience, Bone marrow transplantation, Genetic diseases, Hematopoietic stem cells

## Abstract

Friedreich’s ataxia (FRDA) is a multisystemic neurodegenerative disorder caused by GAA repeat expansion in intron 1 of frataxin (*FXN*), resulting in FXN deficiency and mitochondrial dysfunction. Current therapies do not correct the underlying genetic defect. Here, we demonstrate that ex vivo CRISPR/Cas9 excision of the GAA expansion in hematopoietic stem and progenitor cells (HSPCs) provides durable, systemic therapy for FRDA. A single infusion of gene-edited Sca1^+^ HSPCs, mPPL-001, into myeloablative, humanized YG8JR mice resulted in long-term engraftment of donor-derived cells across hematopoietic and FRDA-affected tissues. mPPL-001 increased FXN expression and corrected multiple key pathogenic drivers of FRDA, including mitochondrial stress, microglial and macrophage activation, and inflammatory signaling. These coordinated effects improved motor functions, preserved cerebellar architecture, mitigated cardiac pathology, and promoted survival of insulin-producing β cells in the pancreatic islets of Langerhans. Collectively, these findings establish *FXN*-edited autologous HSPCs as a one-time, disease-modifying therapy for FRDA and support advancement toward a first-in-human trial.

## Abstract 2

One time infusion of gene edited hematopoeitic stem cells provided durable protection across brain, heart and pancreas in Friedreich’s ataxia mice.

## Introduction

Friedreich’s ataxia (FRDA) is an autosomal recessive, multisystemic disorder primarily caused by homozygous GAA trinucleotide expansion mutation in intron 1 of the frataxin (*FXN*) gene. Affected individuals harbor 44–1,700 GAA repeats, compared with 15–30 in healthy individuals (1). This mutation leads to *FXN* transcriptional repression, reducing protein expression to 5%–35% of normal levels in patients (2). FXN is localized to mitochondria and expressed ubiquitously. While its function is not fully understood, it is essential for iron homeostasis and assembly of iron-sulfur clusters critical for electron transport chain function and ATP production (3). Reduced FXN expression leads to mitochondrial dysfunction, oxidative stress, DNA damage, mitochondrial iron overload, and impaired activity of respiratory complexes (4–7). These pathological changes particularly affect mitochondria-rich organs such as the brain, heart, pancreas, and skeletal muscle.

FRDA is characterized by progressive neurodegeneration in the dorsal root ganglion neurons and spinal cord atrophy, and it extends into dentate nuclei of cerebellum, resulting in gait and limb ataxia, areflexia, and sensory loss in patients (8–13). Disease severity positively correlates with GAA repeat length, and symptoms typically appear between 5 and 15 years of age, with patients becoming wheelchair dependent within 10–15 years of symptom onset (14, 15). Beyond neuropathology, FRDA affects both cardiac and skeletal muscles, with hypertrophic cardiomyopathy being the leading cause of death and skeletal muscle involvement causing weakness, fatigue, and respiratory impairment (16–18). Additionally, diabetes mellitus is a common comorbidity in FRDA and is associated with loss of insulin-producing β cells in pancreatic islets of Langerhans (19).

Currently, omaveloxolone, an NRF-2 (nuclear factor erythroid 2-related factor 2) activator, is the only FDA-approved treatment for FRDA patients aged 16 and older (20). This drug targets oxidative stress pathways and mitochondrial function and may slow the progression of the disease (21, 22) but does not address the root cause of FXN deficiency and therefore requires lifelong, chronic administration to maintain its limited effect (20, 23–25). Gene therapy approaches are being investigated in preclinical and clinical studies and are primarily directed at restoring FXN expression in neurons and cardiomyocytes using adeno-associated virus (AAV) for targeted *FXN* cDNA delivery (26–29). Although encouraging, these approaches have significant limitations: (a) AAV vectors do not enable widespread delivery of therapeutic cargo across multiple organs; (b) achieving systemic delivery to target the CNS and heart requires high doses of AAV vectors, which can result in serious adverse events (30–34); and (c) *FXN* overexpression can be toxic. Indeed, studies on transgenic flies, yeast, and mouse models have shown that FXN overexpression can exacerbate oxidative stress, mitochondrial dysfunction, and cardiac toxicity (35–37). Therefore, a significant unmet need persists for a therapy that can effectively address the multisystemic manifestations of FRDA.

Autologous, gene-modified CD34^+^ hematopoietic stem and progenitor cell–based (HSPC-based) therapies have established an important clinical precedent for durable intervention in inherited multisystemic diseases, including neurodegenerative disorders. In metachromatic leukodystrophy, lentiviral (LV) transduction of autologous CD34^+^ HSPCs with arylsulfatase A (*ARSA*) improved survival and preserved motor and cognitive function beyond the expected natural history of the disease, with therapeutic benefits maintained for greater than 12 years (38), demonstrating long-term durability of this approach. Similarly, in cerebral adrenoleukodystrophy, LV-mediated gene addition of *ABCD1* (ATP-binding cassette subfamily D member 1) into autologous CD34^+^ cells stabilized neurologic function over 24 months (39, 40). More recently, we demonstrated systemic clinical benefit in a phase I/II clinical trial for cystinosis, in which autologous CD34^+^ HSPCs transduced ex vivo with an LV encoding *CTNS* reduced white blood cell cystine levels, decreased cystine crystal burden in skin and intestinal mucosa, and maintained stable neuromotor and cognitive function in patients (41). Therapeutic benefits are mediated by durable HSPC engraftment within the bone marrow niche, sustained multilineage hematopoietic reconstitution, and generation of corrected myeloid progeny that engraft as tissue-resident macrophages in peripheral tissues and microglia-like cells in the CNS, delivering therapeutic proteins through secretion and/or tunneling nanotubes (42–46).

Based on these findings, we applied HSPC transplantation to FRDA and demonstrated that WT HSPCs rescue FRDA pathology in a humanized YG8R mouse model (47). A single systemic infusion of WT HSPCs into lethally irradiated YG8R mice prevented neurologic, muscular, and cardiac manifestations of the disease through engraftment of donor-derived microglia-like cells and macrophages that transferred FXN to the diseased neurons and myocytes. This work established one-time HSPC transplantation as a potentially durable therapy for FRDA that would prevent the multisystemic complications of the disease. Given the risks associated with allogenic stem cell transplantation, we next developed an autologous HSPC gene therapy approach (48). Since *FXN* overexpression is toxic and over 96% of patients with FRDA carry an intronic GAA repeat expansion (49), we designed a CRISPR/Cas9-based strategy to excise the pathogenic repeats (48). Gene-edited, FRDA patient–derived CD34^+^ HSPCs (PPL-001) and lymphoblasts showed elevated *FXN* expression and improved mitochondrial functions. To validate the efficacy of our gene-editing approach in another human, disease-relevant model, a cohort of FRDA patient–derived induced pluripotent stem cells (iPSCs) and isogenic lines, gene edited with our CRISPR/Cas9 approach, were generated (50). GAA excision in FRDA iPSC-derived neurons restored FXN expression and corrected key disease-associated apoptotic, mitochondrial, and ER stress phenotypes (50). Since HSPCs differentiate into microglia-like cells in the CNS (10), the iPSC cohorts were differentiated into iPSC-derived microglia (iMGs). FRDA iMGs exhibited significant mitochondrial defects, abnormal iron accumulation, and a marked pathological inflammatory state that contributed to neuronal death in a coculture model and in Purkinje and granular cell layer (GCL) neurons in a newborn immunodeficient xenotransplantation model (51). Importantly, CRISPR/Cas9 gene editing of the GAA repeat expansion effectively reversed these cellular defects and attenuated the neurodegenerative phenotype (51).

The present study evaluated in vivo efficacy of murine analogous human *FXN* (h*FXN*) gene-edited Sca1^+^ HSPCs, mPPL-001, in the humanized YG8JR mouse model for FRDA. A single systemic infusion of mPPL-001 resulted in durable engraftment of mPPL-001–derived cells in hematopoietic and FRDA-affected tissues, accompanied by multisystemic benefits, including improved motor coordination and preservation of cerebellar, cardiac, and pancreatic integrity. The therapeutic effects were driven by increased hFXN tissue expression and reduced mitochondrial stress and inflammatory signaling. These findings establish the disease-modifying potential of mPPL-001 and support its advancement toward a clinical trial for FRDA.

## Results

### Transplantation of mPPL-001 leads to successful long-term engraftment of mPPL-001–derived cells in hematopoietic compartments and primary organs affected by FRDA.

The therapeutic efficacy of mPPL-001 was assessed in the humanized YG8JR mouse model of FRDA, which harbors an h*FXN* transgene with > 800 GAA repeats on a murine *fxn*-null background (mfxn^–/–^ hFXN^+^) (52). Therefore, this model has the key advantage of enabling the use of our previously optimized h*FXN* gene–editing ribonucleoprotein (RNP) complex (previously called 4RNPEnh) (48), comprising a high-fidelity Cas9 nuclease and 2 sgRNAs (UP4 and DN4) flanking the GAA expansion mutation in h*FXN* and delivered via electroporation in the presence of an electroporation facilitator (48). Genome-wide off-target activity of UP4 and DN4 sgRNAs was assessed by ONE-seq against the hg38 human reference genome, the Human Genome Diversity Project, and variants from the 1000 Genomes Project (Supplemental Figure 1A; supplemental material available online with this article; https://doi.org/10.1172/JCI209546DS1). This analysis nominated 52,109 potential off-target sites for UP4 and 45,998 for DN4 (Supplemental Figure 1A). Subsequent next-generation sequencing of in vitro cleavage products generated using the RNP complex and off-target DNA libraries identified fewer than 5 tier 1 sites (>1% the rate of on-target editing with low functional annotation) and fewer than 71 tier 2 sites (>1% the rate of on-target editing with low functional annotation) and predominantly within intronic/noncoding regions at low frequency of editing, supporting high specificity and a low off-target risk profile for both guides (Supplemental Figure 1B).

To generate the murine surrogate for evaluating the in vivo therapeutic impact of PPL-001, Sca1^+^ bone marrow HSPCs (analogous to human CD34^+^ HSPCs) were isolated from YG8JR mice and edited with RNP complex to produce mPPL-001 or mock electroporated without Cas9 (mock HSPCs) (Figure 1A). Sca1^+^ HSPCs from multiple YG8JR donors were pooled for the generation mPPL-001 and mock HSPCs. Average on-target h*FXN* GAA excision efficiency in Sca1^+^ HSPCs ranged from 7.6% to 30.5%, with a mean of 20.3% (Figure 1B). This variability likely reflects donor-to-donor biological differences of Sca1^+^ HSPCs prior to electroporation. mPPL-001 retained in vitro colony-forming capacity comparable with that of mock HSPCs (Supplemental Figure 1C).

mPPL-001 or mock HSPCs were transplanted into lethally irradiated 2.0- to 2.5-month-old YG8JR recipients (mPPL-001 = 13, 10 male/3 female [10M/3F]; mock HSPC = 11, 6M/5F) and analyzed 6.5–7 months post-transplantation. Age-matched WT (*n* = 9; 6M/3F) and untreated YG8JR (*n* = 9; 5M/4F) littermates served as controls. Engraftment and biodistribution analyses revealed a substantial proportion of mPPL-001–derived cells within the hematopoietic compartments, with mean chimerism level of 7.6% in bone marrow, 5.9% in peripheral blood, 26.5% in the spleen, and 19.8% in the thymus (Figure 1C). Additionally, mPPL-001–derived cells were also detected in multiple FRDA-affected tissues, including the cerebellum (0.52%), cerebrum and brain stem (0.55%), spinal cord (1.09%), heart (2.19%), eye (1.38%), pancreas (3.13%), and skeletal muscle (4.14%) (Figure 1C). The ex vivo h*FXN* GAA excision efficiency in mPPL-001 correlated with the level of mPPL-001–derived cells in the bone marrow (*P* = 0.075; *F* = 3.879; *r*^2^ = 0.26), which in turn correlated with chimerism in spleen (*P* = 0.03; *F* = 6.351; *r*^2^ = 0.36) and thymus (*P* = 0.057; *F* = 4.494; *r*^2^ = 0.29) (Figure 1, D and E). These data demonstrate the durable engraftment of mPPL-001 within the bone marrow niche and its widespread presence across multiple tissues, including those affected by the disease.

### mPPL-001 transplantation leads to increased hFXN expression and improves locomotor function and cerebellar integrity in YG8JR mice.

The humanized YG8JR mice develop progressive locomotor deficits and cerebellar atrophy, beginning around 9 months of age (53). Catwalk gait analysis revealed that untreated and mock HSPC–transplanted YG8JR mice exhibited significant alterations in gait patterns, including prolonged left hind paw swing duration, reduced duty cycle, coupling of hind paws, and imbalanced frequency of cruciate step sequence, cruciate steps A and B, compared with WT mice (Figure 2A). Transplantation of mPPL-001 significantly improved these deficits (Figure 2A) and demonstrated trends of positive correlation between excision efficiency in mPPL-001 and hematopoietic engraftment of mPPL-001 with improvements in left hind swing duration (Supplemental Figure 2A). Histological analysis of cerebellar architecture using H&E staining revealed significant thinning of both the GCL and molecular layer (ML) in untreated and mock HSPC–transplanted YG8JR mice compared with WT controls (Figure 2B). Consistent with these findings, NeuN immunostaining confirmed marked neuronal loss within the GCL of untreated YG8JR and mock mice compared with WT (Figure 2C). Transplantation of mPPL-001 significantly preserved cerebellar integrity, maintaining GCL neuronal density and ML thickness compared with both YG8JR and mock mice, although values remained significantly lower than WT controls (Figure 2, B and C). Notably, GCL and ML thickness was reduced in pretransplant, 2.0- to 2.5-month-old YG8JR mice (age of transplant) compared with WT mice, and mPPL-001 transplantation preserved this architecture at 8 months of age (Figure 2D). These findings indicate that mPPL-001 effectively stabilizes established cerebellar pathology, preventing further progression, although it does not reverse existing damage.

To assess whether improved gait and cerebellar neuroprotection were associated with changes in FXN expression, cerebellar hFXN was evaluated by immunofluorescence. A significant increase in hFXN expression was observed in the cerebellum of mPPL-001 recipients compared with both untreated and mock cohorts (Figure 2E). Cerebellar hFXN expression levels positively correlated with bone marrow engraftment of mPPL-001–derived cells (*P* = 0.04; *F* = 5.28; *r*^2^ = 0.32) (Figure 2F), indicating that higher GAA excision efficiency in HSPCs is associated with increased tissue FXN expression.

The convergence of these effects highlights the importance of defining the level of GAA excision required in the drug product, mPPL-001, for consistent neurological benefit. Gait impairment is the earliest clinical manifestation of FRDA and is seen in all affected individuals (54). This phenotype is recapitulated in the YG8JR mouse model. Cohort-level comparisons among the mPPL-001 recipient YG8JR mice revealed that mPPL-001 with lower editing efficiency of 7.6% conferred limited benefits, whereas cohorts receiving approximately 14% GAA excision showed partial restoration of FXN expression and gait-related measures (Supplemental Figure 3). In contrast, cohorts receiving mPPL-001 with excision efficiencies > 14% showed greater and consistent improvements across YG8JR recipients, suggesting > 14% GAA excision efficiency in mPPL-001 as a minimum threshold for consistent therapeutic benefit in YG8JR mice.

Altogether, these results demonstrate that mPPL-001 transplantation improves locomotor deficits and preserves cerebellar architecture and neuronal integrity in YG8JR mice, with therapeutic effects associated with GAA excision efficiency in HSPCs and increased cerebellar FXN expression.

### mPPL-001 transplantation modulates microglia-mediated cerebellar neuroinflammation in YG8JR mice.

To evaluate the neuroprotective impact of mPPL-001 therapy, bulk RNA-seq was performed on cerebellar tissue at 6 months posttransplant. Principal component analysis revealed that untreated and mock HSPC–transplanted YG8JR mice clustered together and distinctly from WT mice (Supplemental Figure 4A), reflecting disease-associated transcriptional dysregulation. In contrast, mPPL-001–treated mice exhibited a transcriptional profile partially overlapping with WT mice (Supplemental Figure 4A). Permutational multivariate analysis of variance (PERMANOVA) demonstrated a significant effect of mPPL-001 on global transcriptomic composition (pseudo-*F* = 1.74, *R*² = 0.32, *P* = 0.049), indicating that experimental condition accounted for 32% of total transcriptional variance (Supplemental Figure 4B).

To assess transcriptional correction by mPPL-001, we integrated differential expression changes across disease and treatment conditions (Supplemental Figure 4, C and D, and Supplemental Table 1). Of the 709 genes significantly altered with disease and treatment response (*P* < 0.01), 102 were associated with mPPL-001 treatment. This subset included 24 genes that exhibited transcriptional rescue following mPPL-001 compared with disease and 78 genes that were independent of disease signature (Supplemental Figure 4C). Among the rescued genes, 17 were fully restored to WT-like levels, while 7 showed partial recovery. h*FXN* mRNA sequence was manually added to the GRCm39 mouse genome, and its expression was markedly increased in mPPL-001–treated YG8JR mice compared with both untreated and mock HSPC–transplanted mice, although it remained lower than endogenous murine *fxn* levels in WT mice (Figure 3, A and B). Analysis of the rescued gene set revealed enrichment of immune and inflammatory pathways, supporting a targeted therapeutic effect of mPPL-001 on microglia-associated neuroinflammatory programs (Supplemental Table 1). This signature included MHC class II antigen presentation genes (*H2-Aa*, *H2-Eb1*, and the invariant chain Cd74) together with innate immune response receptors (*Tlr3* and *Tlr4*) (Supplemental Table 1). Leading-edge analysis further identified inflammatory and myeloid-associated programs involving *Ikbkg* and *Il15ra*, mononuclear cell recruitment–associated genes (*Marcks* and *Fundc1*) and cell death regulators (*Bcl6* and *Ripk1*) (Figure 3, A and B). These immune-associated changes were accompanied by altered expressions of extracellular matrix and tissue-remodeling transcripts (*Ddr2*, *Hspg2*, *Frem2*, and *Aebp1*) as well as genes associated with membrane signaling, cellular stress response, and metabolic transport (*Cav1*, *Emp3*, *Rcn3*, and *Slc38a5*). Notably, *Xbp1*, the ER unfolded protein response transcription factor that drives inflammatory responses (55), is markedly upregulated in the FRDA disease groups compared with WT, and its expression is normalized in mPPL-001–treated mice (Figure 3B). Together, these findings demonstrate that mPPL-001 transplantation attenuates a coordinated neuroinflammatory program, involving microglial activation, antigen presentation, inflammatory cell death signaling, and extracellular matrix remodeling in the FRDA cerebellum.

GSEA identified significant suppression of transcriptional regulation and genome maintenance programs, DNA integrity checkpoint, and damage response and repair, with representative genes including *Fus*, *Kmt5c*, *Cgas*, *Rnf138*, *Brca2*, *Mcm5*, and *Kdm2a* in untreated and mock HSPC–transplanted YG8JR cerebellum (Figure 3B and Supplemental Table 2). Importantly, these pathways showed partial restoration following mPPL-001 transplantation, demonstrating that mPPL-001 not only attenuates inflammatory programs but also promotes recovery of transcriptional and genomic maintenance pathways that are suppressed in the disease state (Figure 3, A and B, and Supplemental Table 2).

To confirm the microglial and neuroinflammatory transcriptional signatures in YG8JR mice, we analyzed morphology and branching complexity of Iba1^+^ cerebellar microglia by Sholl analysis (Figure 3C). Untreated and mock YG8JR mice displayed amoeboid, activated microglia, whereas mPPL-001 transplantation restored ramified, homeostatic morphology compared with WT mice (Figure 3C).

Together, these findings demonstrate that mPPL-001 transplantation provides cerebellar neuroprotection by modulating the local inflammatory environment, including microglial activation, and normalizing dysregulated transcriptional networks critical for neuronal integrity, providing an additional mechanism for its neuroprotective efficacy in the FRDA disease model.

### mPPL-001 transplantation increases FXN expression in the heart and preserves cardiac architecture.

Cardiac pathology in FRDA involves a complex interplay of mitochondrial proliferation and dysfunction, progressive loss of contractile fibers, fibrosis, and calcium dysregulation (13), resulting in ventricular wall thickening and diastolic dysfunction, while preserving systolic function until advanced stages of the disease (56, 57). Consistent with previous reports describing mild and late-onset cardiac abnormalities in YG8JR mice (10, 58), M-mode echocardiography of untreated 8- to 9-month-old untreated YG8JR mice revealed no significant differences in cardiac function compared with WT controls (Figure 4A). However, myeloablative irradiation is known to exacerbate cellular and mitochondrial stress (59); accordingly, mock HSPC–transplanted YG8JR mice exhibited a restrictive cardiac phenotype characterized by reduced diastolic and systolic left ventricular internal diameter compared with WT (Figure 4A). In contrast, mPPL-001–treated mice exhibited significant improvement in these dimensional parameters, nearing WT levels, and showed positive correlation with engraftment of mPPL-001–derived cells in the hematopoietic compartments (Figure 4A and Supplemental Figure 5, A and B). Elevated left ventricular mass was observed and remained unchanged in YG8JR mice of all cohorts compared with WT mice and was not significantly different among the YG8JR mice (Figure 4A). Notably, this pathology was present in 2-month-old pretransplant YG8JR controls (Supplemental Figure 5C).

Ultrastructural analysis of untreated YG8JR hearts by electron microscopy demonstrated that mitochondrial morphology was comparable with WT, while mock HSPC transplantation significantly increased the proportion of fragmented mitochondria (Figure 4B). In contrast, mitochondrial morphology in mPPL-001 recipients closely resembled WT mice (Figure 4B). Given the subtle mitochondrial phenotype changes in the heart, mitochondrially localized hFXN was quantified using a highly sensitive and previously validated triple quadrupole mass spectrometry method (60–62). Mock mice exhibited significantly reduced mitochondrial hFXN expression compared with untreated YG8JR mice (Figure 4C), consistent with observed structural abnormalities limited to the myeloablative cohort. Notably, mPPL-001 transplantation significantly increased mitochondrial hFXN levels relative to mock mice and showed trends of positive correlation with engraftment of mPPL-001–derived cells within the bone marrow (*P* = 0.1; *F* = 3.2; *r*^2^ = 0.26) (Figure 4D).

In the FRDA patient myocardium, compensatory hypertrophy is associated with chronic, low-grade inflammation, driven by proinflammatory CD68^+^ macrophages (63). Similarly, untreated and mock HSPC–transplanted YG8JR hearts exhibited a significant increase in CD68^+^ macrophages, which were ameliorated in mPPL-001–treated mice (Figure 4E).

These results demonstrate that YG8JR mice, despite exhibiting mild cardiac structural abnormalities, recapitulate key inflammatory pathological features observed in FRDA patients. mPPL-001 transplantation mitigates these abnormalities by increasing mitochondrially localized hFXN expression and attenuating myocardial inflammation, thereby preserving cardiac architecture.

### mPPL-001 transplantation attenuates pancreatic inflammatory macrophages, protecting β cells of the islets of Langerhans in YG8JR mice.

Diabetes affects approximately 10%–30% of FRDA patients and is often associated with impaired glucose tolerance and insulin deficiency (64, 65). In YG8JR mice, fasting blood glucose levels remained comparable with those of WT mice across all groups up to 6 months posttransplantation (Supplemental Figure 6A). Histological analysis of H&E-stained pancreatic sections revealed reduced islet cellularity in untreated and mock HSPC–transplanted YG8JR mice compared with WT controls (Figure 5A). Insulin immunofluorescence confirmed significant loss of β cells in both untreated and mock YG8JR groups compared with WT (Figure 5C). Notably, this islet atrophy was already present in 2-month-old YG8JR mice, which corresponds to the age of mPPL-001 transplant, demonstrating a preexisting condition (Supplemental Figure 6B). Remarkably, mPPL-001 transplantation restored the abundance of insulin-producing β cells to that of healthy controls, demonstrating recovery of physiological architecture (Figure 5, A and B). This restoration of abundance of insulin-positive β cells showed positive correlation engraftment of mPPL-001–derived cells in hematopoietic organs (Supplemental Figure 6C).

Since emerging evidence demonstrates that resolution of CD11b^+^ and CD68^+^ macrophage-mediated pancreatic inflammation can promote β cell regeneration and tissue repair (66–70), we investigated the inflammatory status in YG8JR mice across the treatment groups. Untreated and mock YG8JR mice exhibited significantly increased CD68^+^ inflammatory macrophages in the pancreas compared with WT (Figure 5C). In contrast, CD68^+^ macrophage abundance was significantly decreased in mPPL-001–transplanted mice (Figure 5C). Additionally, mPPL-001 treatment was associated with enrichment of CD206^+^ antiinflammatory macrophages compared with untreated and mock HSPC–transplanted YG8JR mice, nearing WT levels (Figure 5D). Taken together, our results identify an inflammatory component in FRDA pancreatic pathology that could contribute to β cell degeneration and demonstrate that mPPL-001 transplantation exerts therapeutic benefits by supporting a shift toward antiinflammatory immunological modulation and tissue repair in the pancreas and preserving insulin-producing β cells.

## Discussion

FRDA is a multisystemic neurodegenerative disorder that leads to progressive loss of locomotor function, cardiomyopathy, and premature death. Current therapies remain largely supportive, underscoring the urgent need for disease-modifying interventions. Here, we demonstrate that mPPL-001 infusion, the murine analog of a CRISPR/Cas9-mediated gene-edited HSPC product in which the pathogenic GAA expansion in exon 1 of h*FXN* is excised, leads to multiorgan therapeutic benefits in the humanized YG8JR mouse model of FRDA. A single systemic infusion of mPPL-001 established long-term engraftment of gene-edited cells across hematopoietic and disease-affected tissues, with on-target FXN editing efficiencies of 7.6%–30.5% in input cells correlating with bone marrow chimerism. This excision efficiency in HSPCs resulted in increased tissue FXN expression and rescued neurological, cardiac, and pancreatic phenotypes; it was also positively correlated with donor engraftment in the hematopoietic tissues in the YG8JR mice. Together, the sustained multiorgan benefit observed at terminal analysis demonstrates long-term durability of mPPL-001 following a single systemic administration and provides, to our knowledge, the first evidence that autologous, gene-modified CD34^+^ HSPC-based approaches can effectively target a disorder driven by a mitochondrial protein deficiency, highlighting a promising new treatment paradigm for FRDA. These findings support the potential for PPL-001 to address the systemic pathology of FRDA, a major limitation of viral vector–based gene therapies (36, 71, 72).

Neurologically, FRDA manifests as progressive gait and balance impairments, dysarthria, and cerebellar dysfunction (8–13). YG8JR mice recapitulate key neuropathological features of the disease, including degeneration of the cerebellar GCL, ML thinning, cerebellar inflammation, and early microglial activation (73–77). Myeloablative preconditioning facilitated widespread engraftment of mPPL-001–derived cells in YG8JR brain, including the cerebellum, leading to increased FXN expression, preservation of the cerebellar neuronal integrity, and amelioration of gait impairment in the YG8JR mice. We previously showed that FXN-GFP was transferred from the transplanted HSPC-derived microglia-like cells to the diseased neurons, leading to their preservation (47). In this study, transcriptomic profiling revealed that mPPL-001 acts through multiple complementary mechanisms, including reengaging mitochondrial stress signaling, attenuating maladaptive neuroimmune signaling, and restoring cellular homeostasis with the YG8JR cerebellum. mPPL-001 transplantation restored expression of key components of mitochondrial quality control machinery, including mitophagy regulators (*Pink1*, *Fis1*, and *Parl*) and autophagy-associated genes (*Dapk2*, *Rin3*, *Alox5*, *Sirpa*, and *Fundc1*), which coordinate mitochondrial health, fragmentation, and clearance (78–81). Interestingly, we observed normalization of *Xbp1*, a key regulator of ER stress response and mitophagy (82), in mPPL-001–transplanted YG8JR mice. We previously demonstrated a similar correction in iPSC-derived, *FXN* gene–edited FRDA neurons compared with FRDA neurons, in which severe ER structural damages and reduced ER-mitochondrial contact were restored following *FXN* correction (50). These findings support an emerging role for ER stress pathways in FRDA neuronal dysfunction and indicate that mPPL-001–mediated restoration of FXN can modulate this pathology. Reactivation of these mitochondrial quality control pathways in mPPL-001–transplanted mice likely enhances the removal of damaged mitochondria and alleviates oxidative and bioenergetic stress, thereby contributing to a neuroprotective environment.

In parallel, mPPL-001 also modulated inflammatory and microglial pathways in YG8JR cerebellum. Emerging evidence indicates that microglial dysregulation contributes to FRDA neuropathology, with both patient tissues and mouse models showing microglial activation and inflammatory remodeling in the cerebellum (83–86). More recently, FXN deficiency was shown to perturb microglial homeostasis in FRDA iMGs, driving a hyperactivated, proinflammatory phenotype characterized by mitochondrial and lysosomal defects and imbalanced iron, lipid, and ROS homeostasis (51). FRDA iMGs exerted direct neurotoxic effects, inducing neuronal death in vitro and contributing to degeneration of Purkinje and GCL neurons in vivo (51). Importantly, excision of the pathogenic GAA repeats (using the same CRISPR components as mPPL-001) and restoring FXN expression markedly attenuated these pathological phenotypes (51). These findings support a therapeutic mechanism in which *FXN* correction within the microglial lineage can mitigate neurodegeneration and is reflected by the neuroimmune changes observed in cerebella of mPPL-001 recipients. Normalization of antigen presentation genes (*H2-Aa*, *H2-Eb1*, and *Cd74*) together with innate immune receptors (*Tlr3* and *Tlr4*) indicates attenuation of disease-associated inflammatory programs. Additional correction of *Ripk1*, *Bcl6*, *Marcks*, and *Fundc1* further supports attenuation of inflammatory signaling, immune cell migration, and mitochondrial regulation of immune activation (87–92). The transcriptional changes were accompanied by transition of microglial morphology toward a homeostatic, ramified phenotype in mPPL-001 recipients. The widespread morphological normalization within Iba1^+^ populations suggests a significant contribution of engrafted cells to an overall decrease of microglial activation. This microglial remodeling parallels findings in the 5xFAD mouse model of Alzheimer’s disease, where WT HSPC transplantation led to global suppression of microgliosis and neuroinflammation, decrease of amyloid plaques, and preservation of the blood–brain barrier, collectively improving neurocognitive functions (93).

mPPL-001 also reengaged transcriptional programs linked to genotoxic stress. Interestingly, FXN deficiency was shown to drive oxidative DNA damage and impair DNA repair pathways in patients (94, 95). We and others have shown that FXN deficiency induces microglial DNA damage (51, 96, 97). In parallel, expanded GAA repeats are associated with replication stress, DNA-RNA hybrid formation, and repeat instability (98, 99). Epigenetic changes, including altered DNA methylation and repressive chromatin marks, were reported in patients with FRDA, and epigenetic modulators have been explored as a potential therapeutic strategy in preclinical and clinical studies (100–103). In our study, YG8JR mice recapitulated these key signatures. Notably, mPPL-001 transplantation partially restored these pathways, including DNA damage response, double-strand break repair, recombinational repair, DNA replication, and epigenetic regulation. Overall, mPPL-001 transplantation increased FXN protein expression, reengaged mitochondrial quality control and genome-maintenance pathways, and suppressed maladaptive microglial inflammatory signatures, thereby providing multiple complementary mechanisms that foster a neuroprotective microenvironment in FRDA cerebellum. These combined effects contributed to preserved cerebellar architecture and circuitry and improved motor performance in YG8JR mice.

Cardiac manifestations in FRDA, characterized by hypertrophic cardiomyopathy and diastolic dysfunction, represent the leading cause of mortality (18, 56, 104). YG8JR mice reproduce key features of this pathology, exhibiting early-onset left ventricular hypertrophy that persists into adulthood, although the overall cardiomyopathic manifestations remain mild (10, 58, 105). Notably, mPPL-001 transplantation did not reverse the preexisting hypertrophy in YG8JR mice, indicating that HSPC therapy stabilized established cardiac pathology rather than reversing the structural remodeling. This stabilization effect is associated with improved ventricular geometry, increased mitochondrial FXN expression, and reduced macrophage-driven myocardial inflammation. The increase in mitochondrial FXN was observed relative to mock HSPC–transplanted mice, consistent with the exacerbated pathological remodeling in this group because of myeloablation and demonstrate that even modest restoration of FXN expression is sufficient to preserve cardiac integrity. This aligns with previous studies showing that approximately 17% of WT FXN levels is sufficient for preserving cardiac function (106, 107).

FXN deficiency drives senescence and apoptosis of insulin-producing β cells, resulting in loss of β cell mass in FRDA mouse models and patient autopsy samples (19, 108, 109). In YG8JR mice, we observed early and persistent islet deterioration, with significant β cell depletion already evident at 2 months of age, prior to transplantation. Importantly, to our knowledge, our study reveals for the first time that FRDA pancreatic degeneration is accompanied by a significant inflammatory component, characterized by accumulation of proinflammatory CD68^+^ macrophages in the pancreas. Inflammatory macrophages are potent mediators of β cell injury, releasing cytokines that suppress insulin secretion, activate oxidative and ER stress pathways, and exacerbate β cells loss that are intrinsically vulnerable due to their low antioxidant capacity (110, 111). Indeed, increased infiltration of CD68^+^ and CD11c^+^ macrophages within and around the islets is a hallmark of type 2 diabetes pathology (112) that amplifies metabolic stress. Similarly, studies in autoimmune type 1 diabetes have demonstrated that islet-resident macrophages act as early initiators of β cell destruction, and their depletion in murine models markedly reduces diabetes incidence (113), highlighting the causal role of macrophage-driven inflammation in β cell loss. mPPL-001 transplantation markedly reduced proinflammatory CD68^+^ macrophage population within the YG8JR pancreas, alleviating this inflammatory burden. Treatment was associated with enrichment of antiinflammatory CD206^+^ macrophages, restoring a microenvironment that supports β cell survival and proliferation (114), as evidenced by the significantly increased abundance of insulin-producing β cells compared with FRDA disease groups. mPPL-001 also exhibited potential regenerative response, as indicated by the rescue of severe β cell depletion present in 2-month-old FRDA mice. Thus, in contrast to the stabilization observed in the heart and cerebellum, where cardiomyocytes and neurons are predominantly postmitotic, the pancreas showed evidence of tissue-specific reparative and regenerative potential. Together, these findings reveal a tissue-specific reparative capacity of mPPL-001 within the FRDA pancreas.

Collectively, our results establish mPPL-001 as a systemic therapeutic candidate for FRDA that acts on multiple convergent drivers of disease pathogenesis. By restoring FXN expression, reprogramming pathological inflammatory signaling in microglia and macrophages, and promoting tissue-specific repair mechanisms, mPPL-001 achieves robust phenotypic rescue across the nervous system, heart, and pancreas. These findings provide compelling preclinical evidence that PPL-001 can exert disease-modifying potential for FRDA and strongly supports its advancement toward clinical translation. More broadly, they establish a proof of concept for new therapeutic avenues for genetic disorders involving mitochondrial proteins.

## Methods

### Sex as biological variable

Both male and female mice were included in the study to evaluate the therapeutic benefits of mPPL-001.

### ONE-seq off-target analysis

The ONE-seq assay was conducted by SeQure Dx (MaxCyte), with modifications from a previously published protocol (115). Candidate UP4 and DN4 sgRNA off-target sites were designed using Guide Profiler, a proprietary, in silico algorithm allowing up to 6 differences (nucleotide mismatches and up to 2 insertions/deletions) against the hg38 human reference genome, the 1000 Genomes project (116), and the Human Genome Diversity Project (117). A custom oligonucleotide library (Agilent Technologies) was PCR amplified, AMPure XP purified (Beckman Coulter), and used for in vitro cleavage. Cas9:gRNA RNPs were formed at a 1:2 molar ratio (25 nM Cas9 and 50 nM gRNA) and incubated with a DNA library at RNP/DNA ratios of 10:1, 1:1, and 0.1:1, in triplicates, at 37°C for 2 hours. Reactions were quenched with Proteinase K (NEB), purified, end repaired, A tailed, adapter ligated, size selected using PippinHT (Sage Science), PCR amplified, and sequenced on an Illumina NextSeq 2000 instrument. ONE-seq analysis was performed as previously described, with proprietary modifications. A ONE-seq score, defined as the ratio of off-target to on-target read counts and reflecting in vitro cleavage efficiency, was assigned to each nominated off-target site. Candidate off-target sites were ranked based on the ONE-seq score alongside the genomic annotation.

### Mouse model

Therapeutic efficacy was evaluated in the YG8JR mouse model of FRDA, which harbors an h*FXN* transgene with > 800 GAA repeats on a murine *fxn-*null background. Mice were generated in-house by breeding heterozygous h*FXN* carriers with heterozygous h*FXN* noncarriers or obtained from The Jackson Laboratory (strain 030395) (118). WT littermates from the breeding colony served as controls. YG8JR mice were randomly categorized as untreated, mock, or mPPL-001 recipients. All mice were housed in a temperature- and humidity-controlled animal facility with a 12-hour light/dark cycle and had ad libitum access to food and water.

### HSPC isolation, gene editing, and transplantation

#### HSPC isolation.

Bone marrow cells were flushed from femurs and tibias of 8- to 12-week-old YG8JR mice. Sca1^+^ HSPCs were isolated by immunomagnetic separation using anti-Sca1^+^ antibody (catalog 130-123-124, Miltenyi Biotec) and incubated at 37°C and 5% CO_2_ in StemSpan medium (catalog 9600, Stem Cell Technologies) supplemented with murine IL-3 (200 ng/mL), murine SCF (100 ng/mL), murine FLT3L (100 ng/mL), and murine TPO (100 ng/mL).

#### Gene editing.

HSPCs were electroporated using the Neon transfection system (Thermo Fisher Scientific). mPPL-001 was generated by electroporating 1.1 × 10^6^ cells in 90 μL buffer T with 1.8 μM Alt-R-Cas9 electroporation enhancer (catalog 1075915, IDT) and 10 μL RNP solution containing 3 μL SpyFi Cas9 Nuclease (catalog 9214-0.25MG, Aldevron), 11 μM each of sgRNA UP4 and sgRNA DN4 (Synthego Corporation), and 2 μL buffer T. HSPCs were electroporated at 1,700 V (1 pulse, 20 ms), plated at 4.0 × 10^6^ cells/mL density in StemSpan medium (supplemented with 100 ng/mL murine SCF and 200 ng/mL murine TPO), and incubated for 24 hours. Mock HSPCs were electroporated similarly without Cas9.

#### Transplantation.

2.0- to 2.5-month-old recipient YG8JR mice were lethally irradiated (7 Gy; X-Rad 320, PXi) 24 hours prior to stem cell infusion. Mice were injected with 1.5–1.75 × 10^6^ mock HSPCs or mPPL-001 cells per mouse via tail vein injection. A subset of mock HSPCs and mPPL-001 was retained in culture for 7 days to evaluate the on-target h*FXN* GAA excision efficiency. Mice were maintained on antibiotic water containing sulfamethoxazole and trimethoprom (oral suspension) for 1 month after transplant to prevent post-myeloablation infection.

#### Colony-forming unit assay.

15,000 Sca1^+^ HSPCs obtained from WT, mock HSPCs, and mPPL-001 produced using the above steps were cultured in MethoCult GF (catalog M3434, StemCell Technologies) for 12 days in 35 mm dishes. All colonies across conditions were imaged using an AWG EVOS microscope (Thermo Fisher Scientific). The colonies were identified (119) and counted by an unbiased assessor.

### In-life monitoring

#### Periodic blood glucose measurement.

Mice were abstained from food for a minimum of 7–8 hours. Blood glucose levels (mg/dL) were assessed with a drop of blood from the tail and a CVS Health Advanced Glucose Meter.

#### Catwalk gait analysis.

Gait analysis was conducted prior to euthanizing the mice. Mice were acclimated in the testing room for 30 min, and gait analysis was conducted using the CatWalk system (Nodulus Instruments), where mice walked across an illuminated glass walkway, capturing paw prints. A minimum of 3 uninterrupted runs at consistent speed were analyzed per mouse.

#### Echocardiography.

Mice were anesthetized using 1.5% inhaled isoflurane, and 2D M-mode echocardiography was performed with Vevo 2100 from FUJIFILM VisualSonics at the UCSD Seaweed Canyon Cardiovascular Physiology Laboratory by an unbiased technical expert.

### Histopathology

Following euthanasia, dissected tissues were fixed in 10% neutral buffered formalin for 24 hours and transferred to 70% ethanol. Samples were processed for paraffin embedding, sectioning, and H&E staining at the UCSD Moores Cancer Center Biorepository and Tissue Technology Center. Images were captured using a Keyence BZX-800 microscope and analyzed with ImagePro software, version 10.0 (Media Cybernetics).

### Electron microscopy

Fresh left ventricular heart tissue was processed for electron microscopy at the UCSD Cellular & Molecular Medicine Electron Microscopy Core. Biopsies were fixed in 2% paraformaldehyde/2.5% glutaraldehyde in 0.15 M sodium cacodylate buffer (pH 7.4), post-fixed with 1% osmium for 1–2 hours on ice, dehydrated through graded alcohol, and embedded in Durcupan (catalog 44610-1EA, Sigma-Aldrich). 60 nm ultrathin sections were cut on a Leica microtome, stained with uranyl acetate and lead, and imaged on a JEOL 1400 Plus transmission electron microscope at 80 kV with a Gatan 4k × 4k camera. Mitochondrial structure was analyzed using ImagePro software. Mitochondria with area < 0.5 μm^2^ were classified as fragmented (120, 121). Classification was performed on 5–7 randomly selected fields and 35–50 mitochondria per animal by a blinded assessor.

### Droplet digital PCR for gene-editing efficiency

Efficiency of on-target h*FXN* gene editing in mPPL-001 and engraftment of mPPL-001–derived cells across tissues were measured using previously optimized primers and droplet digital PCR (ddPCR) protocol (48). Genomic DNA was isolated from 7-day Sca1^+^ HSPC cultures with Quick Extract (catalog QE0905T, LGC Biosearch Technologies) and from tissues using the DNeasy Blood and Tissue Kit (catalog 69505, Qiagen). The PCR reaction contained 100 ng genomic DNA, 20 U HindIII-HF (catalog R3104S, New England Biolabs), 2× ddPCR Supermix for probes (no dUTP; catalog 1863024, Bio-Rad), and previously described primers (48): *FXN* intron 1 forward: GGTTGCATTTACACTGGCTTC, reverse: AGAGAAGTGACAAGCATGGAG, probe: AGTCGCACCGCAGGACAAAATG; and GAA excision forward: GAAGATAAAGGTGACGCCCA, reverse: GGGTTATCATGGGAGTGAAACT, probe: TTGCGGACCTGGTGTGAGGA. Droplets were generated using Droplet Generation Oil for Probes (Bio-Rad) on the QX200 droplet generator and PCR amplified using the following cycling conditions: 95°C for 10 min ramp at 2°C/s, 94°C for 30 s ramp at 2°C/s, 60°C for 1 min ramp at 2°C/s (×39), and 98°C for 10-min ramp at 2°C/s. Plates were read on a QX200 Droplet reader (Bio-Rad) and analyzed using QuantaSoft software (version 1.7.4.0917). Data are presented as percentages of GAA excision over reference gene on h*FXN*.

### Immunofluorescence

Paraffin-embedded 5 μm tissue sections were deparaffinized with xylene, rehydrated with alcohol gradient, subjected to Tris-EDTA antigen retrieval, and permeabilized with washing buffer containing 0.1% Tween 20 in 1× TBS (TBST; pH 7.4). Sections were blocked with 3% BSA in 1× TBST and incubated overnight at 4°C with the following primary antibodies: anti-FXN (catalog LS-B15909-50, LS-Bio), anti-insulin (catalog 66198-1-Ig, Proteintech), anti-Iba1 (catalog 234-017, Synaptic Systems), anti-NeuN (catalog 702022, Thermo Fisher Scientific), anti-CD68 (catalog 97778S, Cell Signaling Technology), and anti-CD206 (catalog 76220, Cell Signaling Technology). They were then visualized with Alexa Fluor Plus secondary antibodies (catalog A32795, A32773, A48270, and A32790, Thermo Fisher Scientific). Whole tissue sections were stitched on a Keyence BZX-800 microscope and analyzed with ImagePro and ImageJ software.

### FXN mass spectrometry

Mature mitochondrial hFXN was quantified at the Blair lab, University of Pennsylvania, using a previously published protocol (61). Briefly, flash-frozen heart tissue samples were homogenized in 1 mL lysis buffer (RIPA buffer with 0.25% NP-40, 150 mM NaCl, 20 mM Tris-HCl, 5 mM EDTA, protease inhibitor, and 0.01% 2-mercaptoethanol) using a Bullet Blender 24 Gold tissue homogenizer (Next Advance) at 4°C. SILAC-labeled FXN-M protein was spiked into sonicated homogenate and incubated overnight with FXN monoclonal antibody–conjugated dynabeads at 4°C. Eluted protein was dried under nitrogen flow, resuspended in 25 nM aqueous NH_4_HCO_3_ solution containing trypsin, digested at 37°C for 18 hours, and analyzed by UHPLC-MRM/MS using an Agilent 1290 Infinity II UHPLC system interfaced with a 6495C triple quadruple mass spectrometer (Agilent Technologies).

### Bulk mRNA sequencing and analysis

mRNA was isolated from flash-frozen cerebellum with a PureLink RNA Mini Kit (catalog 12-183-018A, Thermo Fisher Scientific). RNA quality was assessed using the Agilent Tapestation 4200, and 50 ng of rRNA-depleted RNA with RNA integrity number greater than 7.0 was used to generate Illumina stranded mRNA libraries. The libraries were multiplexed and sequenced with paired-end reads (PE150) to a depth of approximately 25 million reads per sample on a NovaSeq X Plus (Illumina) and demultiplexed using bcl2fastaq conversion software (Illumina).

FASTQ files were aligned to the GRCm39 mouse genome supplemented with h*FXN* sequence using STAR quantification mode, and count matrices were analyzed in R. Differential gene expression analysis was performed using DESeq2 and ComBat for batch correction.

Global transcriptomic differences were assessed using PERMANOVA implemented in vegan (adonis2 function) (122). Euclidean distance matrices were computed from variance-stabilized expression values generated by DESeq2. Statistical significance was determined using 999 permutations. Global PERMANOVA was performed to evaluate overall group effects on multivariate transcriptomic structure. Pairwise PERMANOVA comparisons were subsequently conducted, and *P* values were adjusted for multiple testing using the Benjamini-Hochberg FDR. Effect sizes are reported as *R*² values representing the proportion of variance explained by each factor, and pseudo-*F* statistics were used to estimate relative between-group separation.

To comprehensively assess disease, procedural, and therapeutic effects, differential expressions were evaluated across all biologically relevant pairwise comparisons. Genes were considered significantly differentially expressed at adjusted *P* value < 0.05 unless otherwise specified. For rescue-pattern analyses designed to detect coordinated but modest therapeutic shifts in the setting of biological heterogeneity, a threshold of *P* < 0.01 and |log_2_ fold change| > 0.5 was applied.

To identify transcripts exhibiting treatment-associated reversal of disease dysregulation, directional rescue analysis was performed. Genes significantly dysregulated in untreated versus WT were classified as disease-associated transcripts. These genes were then evaluated in the mPPL-001 versus untreated comparison, and genes demonstrating significant opposite-direction fold changes were classified as rescued transcripts. Rescued genes that were no longer significantly different in the mPPL-001 versus WT comparison were further categorized as fully rescued, whereas those remaining significantly altered relative to WT were considered partially rescued.

Genes were ranked by the metric −log_10_(adjusted *P* value) × log_2_ fold change and were analyzed using the Gene Set Enrichment Analysis software (v4.4.0; https://www.gsea-msigdb.org/gsea/index.jsp) with the preranked module, employing enrichment statistics and the “max probe” option for collapsing. Leading-edge analysis was performed on selected pathways, and the most distinct genes were visualized using *z* scores. 

### Sholl analysis

Microglial morphology was analyzed using Imaris 3D Image Analysis software in filament reconstruction mode and with images obtained from 20–25 Iba1^+^ microglia in the cerebellum. Microglial surface was reconstructed; fused cells were manually separated or excluded when separation was not feasible. Data were exported were further analysis.

### Statistics

No animals were excluded from the study. To the extent possible, experimenters were blinded to the genotype and treatment conditions of the mice. Blinding was not applied to automated, nonobjective computational measurements. Statistical analysis and graph generation were performed using GraphPad Prism version 10.0. Group comparisons were conducted using 1-way ANOVA followed by Tukey’s/Dunnett’s multiple comparisons or 2-way ANOVA. For data that were not normally distributed, Kruskal-Wallis test followed by Dunn’s multiple comparison was used. Data are presented as mean ± SEM. *P* values of less than 0.05 were considered significant.

### Study approval

Mice were maintained in accordance with UCSD research guidelines for the care and use of laboratory animals, and all procedures were approved by the IACUC (study number S12288).

### Data availability

RNA-seq data generated in this study are deposited in the Gene Expression Omnibus under accession number GSE326123. Custom R scripts for directional analysis, GSEA pathway visualization, and leading-edge heatmap generation are available at https://github.com/cherquilab/fa-rnaseq-rescue/1397645907 (commit ID e6b245514eaef3fb008ec5dd0d7ad796209dab92).Values for all data points in graphs are reported in the Supporting Data Values file.

## Author contributions

SC and AS have full access to all data in the study and take responsibility for its integrity and accuracy. Concept and design: SC and AS. Acquisition, analysis, or interpretation of data: AS, ANC, KJM, ABS, RM, LREH, DPP, IAB, TR, CM, XW, JNR, RABG, VK, KET, KB, and SC. RNA-seq analysis: BBG. Drafting of the manuscript: SC, AS, and BBG. Statistical analysis: AS, SA, BBG, and SC. Funding acquisition: SC.

## Conflict of interest

SC is confounder, shareholder, and member of both the Scientific Board and Board of Directors of Papillon Therapeutics Inc. SC serves as a member of the Scientific Review Board and Board of Trustees of the Cystinosis Research Foundation. This work is covered under the patent entitled “Methods for Treating Mitochondrial Disorders” (number 114198-3029). AS is a paid consultant for Papillon Therapeutics Inc. The terms of this arrangement have been reviewed and approved by the University of California, San Diego in accordance with its conflict-of-interest policies.

## Funding support

This work is the result of NIH funding, in whole or in part, and is subject to the NIH Public Access Policy. Through acceptance of this federal funding, the NIH has been given a right to make the work publicly available in PubMed Central. This work is the result of California Institute of Regenerative Medicine (CIRM) funding, representing the IND-enabling preclinical studies for PPL-001.

- CIRM grants TRAN1-13983 and PDEV-19140 to SC.
- NIH grants R01NS108965, R01NS135162, and R01AG086443 to SC.
- NIH grants NS047101, OD030505, and OD036455 to UCSD Neuroscience Microscopy Shared Facility.
- NIH grant P30 CA 23100 to UCSD Moores Cancer Center.
- NIH shared instrumentation grant S10 OD026929 to UCSD IGM Genomics Center.
- Friedreich’s Ataxia Research Alliance grant to SC.
- Uplifting Athletes grant to AS.
- The Cystinosis Research Foundation grants to SC.
- CIRM fellowships EDUC2-08388 and EDUC2-12611 to KJM and ABS, respectively.

## Supplementary Material

Supplemental data

Supplemental table 1

Supplemental table 2

Supporting data values

## Figures and Tables

**Figure 1:**
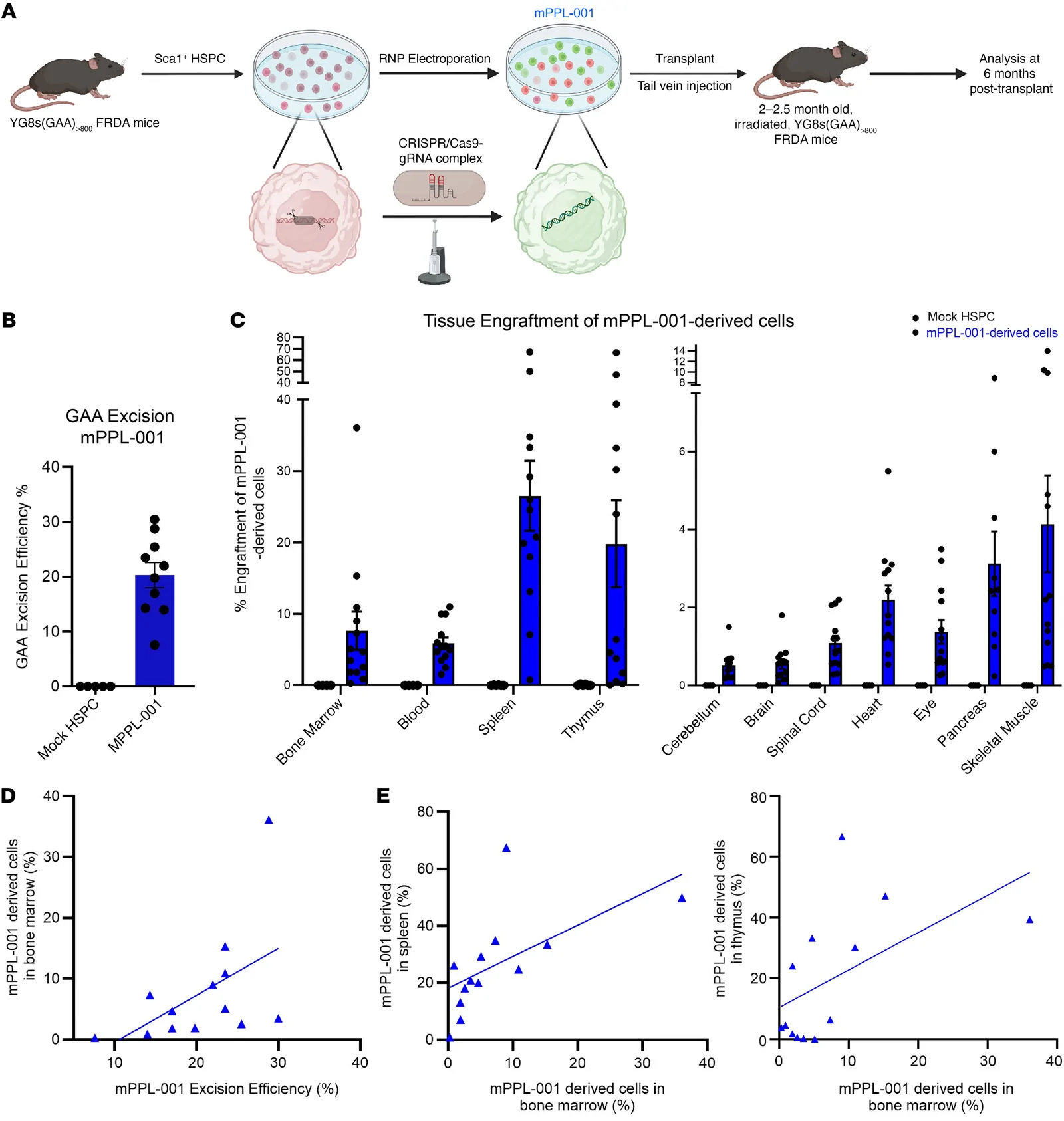
mPPL-001–derived cells engraft in hematopoietic and FRDA-affected tissues in YG8JR mice. (**A**) Schematic of generation and transplantation of mPPL-001 (created with BioRender). (**B**) h*FXN* GAA excision efficiency in mPPL-001 assessed 7 days after electroporation (mock HSPCs, *n* = 5; mPPL-001, *n* = 10). (**C**) Engraftment of mPPL-001–derived cells in hematopoietic tissues and organs affected by FRDA (mock = 2M/2F and mPPL-001= 10M/3F for all tissues except cerebellum [*n* = 12] and pancreas [*n* = 10]). (**D**) Regression analysis of excision efficiency in mPPL-001 versus mPPL-001–derived cells engrafted in bone marrow (*P* = 0.075; *F* = 3.879; *r*^2^ = 0.26). (**E**) Regression analysis of mPPL-001–derived cells in bone marrow versus engraftment in spleen (*P* = 0.03; *F* = 6.351; *r*^2^ = 0.36) and thymus (*P* = 0.057; *F* = 4.494; *r*^2^ = 0.29). Data are presented as mean ± SEM.

**Figure 2:**
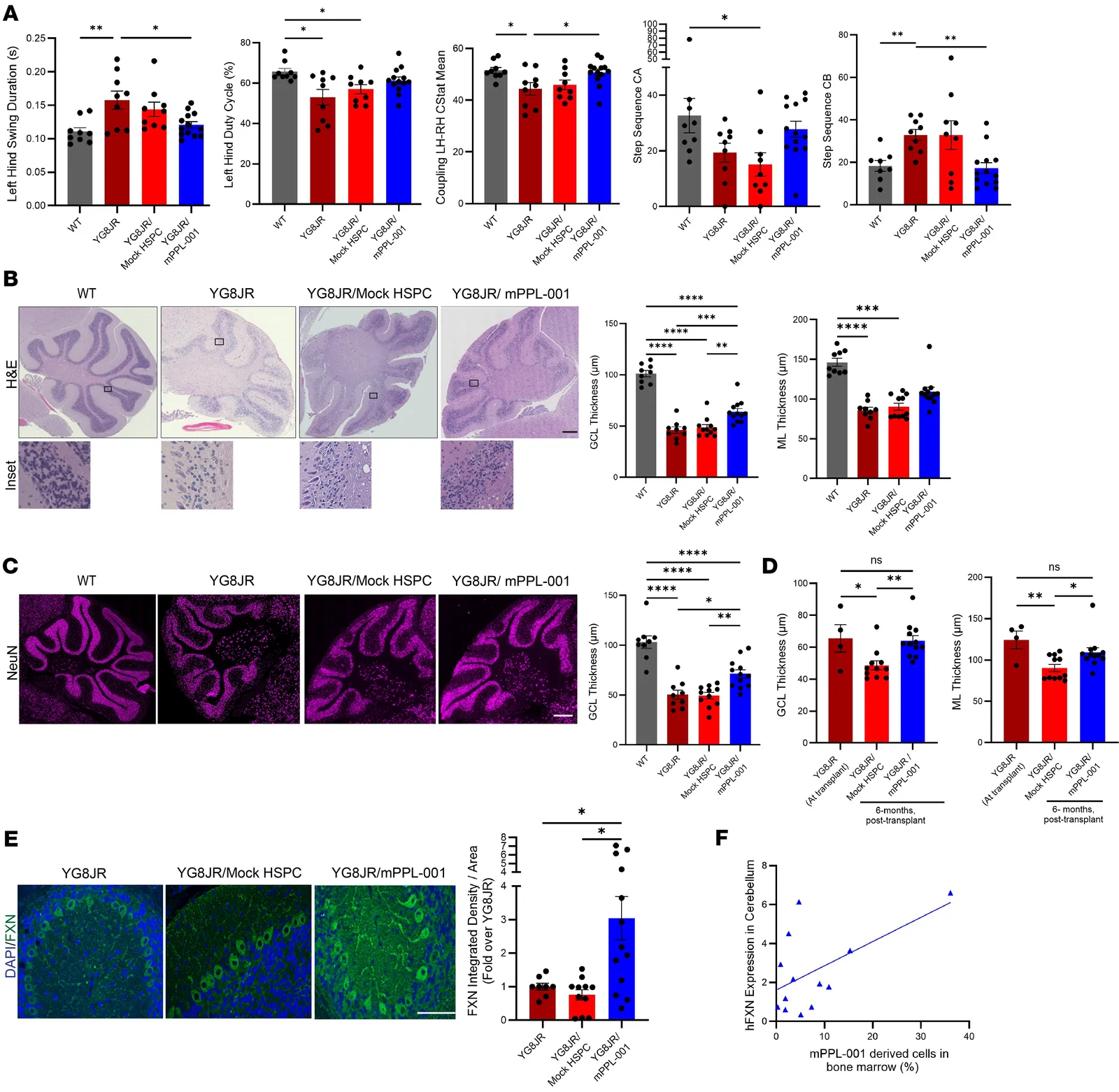
Transplantation of mPPL-001 preserves gait performance and cerebellar architecture in YG8JR mice. (**A**) Catwalk gait parameters in YG8JR mice transplanted with mPPL-001 (YG8JR/mPPL-001) compared with untreated (YG8JR) and mock HSPC–transplanted (YG8JR/mock HSPC) YG8JR controls and WT littermates (WT). One mock HSPC recipient was excluded from analysis owing to weak paw prints and noncompliant runs (WT = 6M/3F, YG8JR = 5M/4F, mock = 5M/4F, and mPPL-001 = 10M/3F). (**B**) Representative H&E cerebellar images and quantification of GCL and ML thickness (WT = 6M/3F, YG8JR = 5M/4F, mock = 6M/5F, and mPPL-001 = 10M/3F) (**C**) Representative NeuN images of cerebellum and quantification of GCL thickness. (**D**) GCL and ML thickness in 2.0- to 2.5-month-old YG8JR mice (age at transplant) and 8-month-old mPPL-001 recipients. (**E**) Representative images and quantification of hFXN immunostaining. Scale bars: 50 μm. (**F**) Regression analysis of bone marrow engraftment versus cerebellar hFXN expression in mPPL-001 recipients. Data are presented as mean ± SEM, analyzed by 1-way ANOVA. *P ≤ 0.05, **P ≤ 0.01, ***P ≤ 0.001, and ****P ≤ 0.0001.

**Figure 3:**
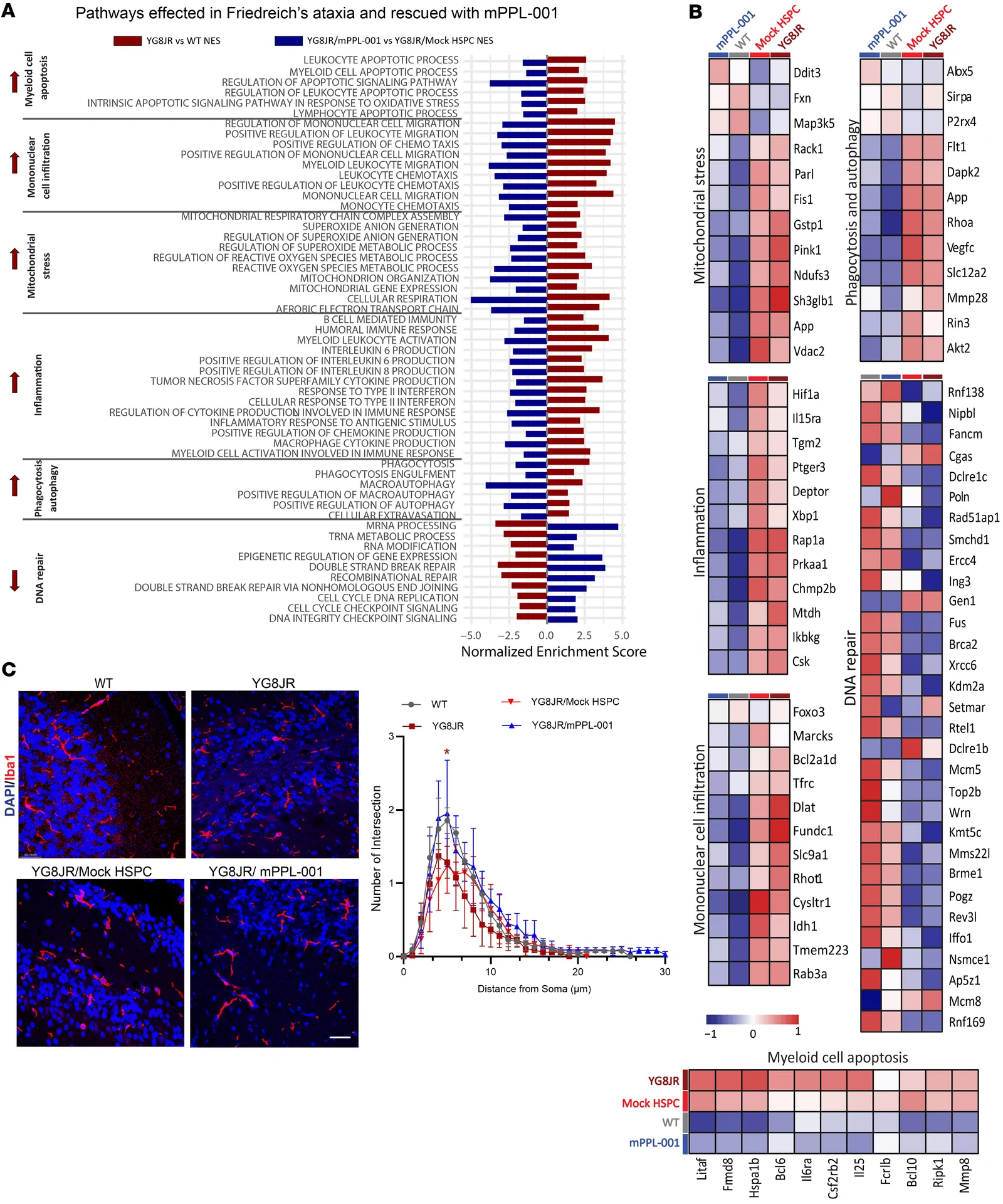
mPPL-001 transplantation partially restores the transcriptomic profile of YG8JR cerebellum, improving microglial stress and neuroinflammation. (**A**) Pathway enrichment analysis of key drivers of cerebellar pathology in YG8JR mice (WT = 2M/1F, YG8JR = 2M/2F, mock = 2M/2F, and mPPL-001 = 4M/1F). (**B**) Leading-edge analysis of genes contributing most to pathway enrichment. (**C**) Representative images of cerebellar Iba1^+^ microglia and Sholl analysis of ramifications. Statistical analysis compares number of intersections among the YG8JR group, and the asterisk represents significance between mock HSPC and mPPL-001 YG8JR recipients. Scale bar: 50 μm. Data are presented as mean ± SEM, analyzed by 1-way ANOVA. *P ≤ 0.05.

**Figure 4:**
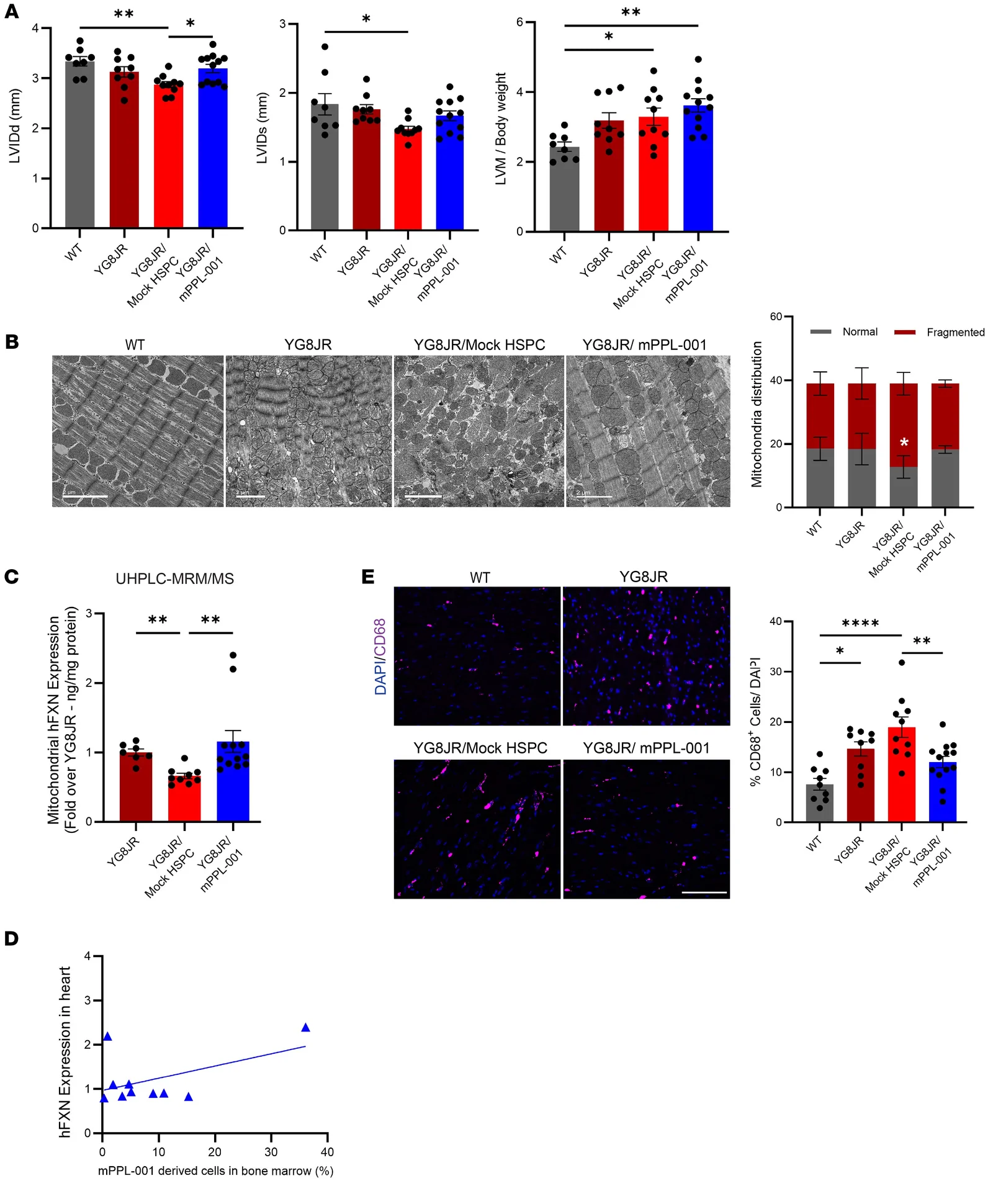
mPPL-001 transplantation improves cardiac architecture and inflammation in YG8JR mice. (**A**) M-mode echocardiography parameters in mPPL-001–transplanted mice compared with controls (WT = 5M/3F, YG8JR = 5M/4F, mock = 6M/4F, and mPPL-001 = 9M/3F). LVIDd, diastolic left ventricular internal diameter; LVIDs, systolic left ventricular internal diameter; LVM, left ventricular mass. (**B**) Representative electron microscopy images and quantification of mitochondrial morphology in heart sections. Scale bars: 2 μm. The asterisk represents significance of normal versus fragmented mitochondria distribution in mock HSPC recipients. (**C**) Mature mitochondrial hFXN expression in YG8JR mice. (**D**) Regression analysis of mPPL-001–derived cells in bone marrow versus mitochondrial hFXN expression in mPPL-001 recipients. (**E**) Representative immunofluorescence images and quantification of CD68^+^ macrophages in the heart. Scale bar: 50 μm. Data are presented as mean ± SEM, analyzed by 1-way (**A**, **C**, and **E**) or 2-way (**B**) ANOVA. *P ≤ 0.05, **P ≤ 0.01, and ****P ≤ 0.0001.

**Figure 5:**
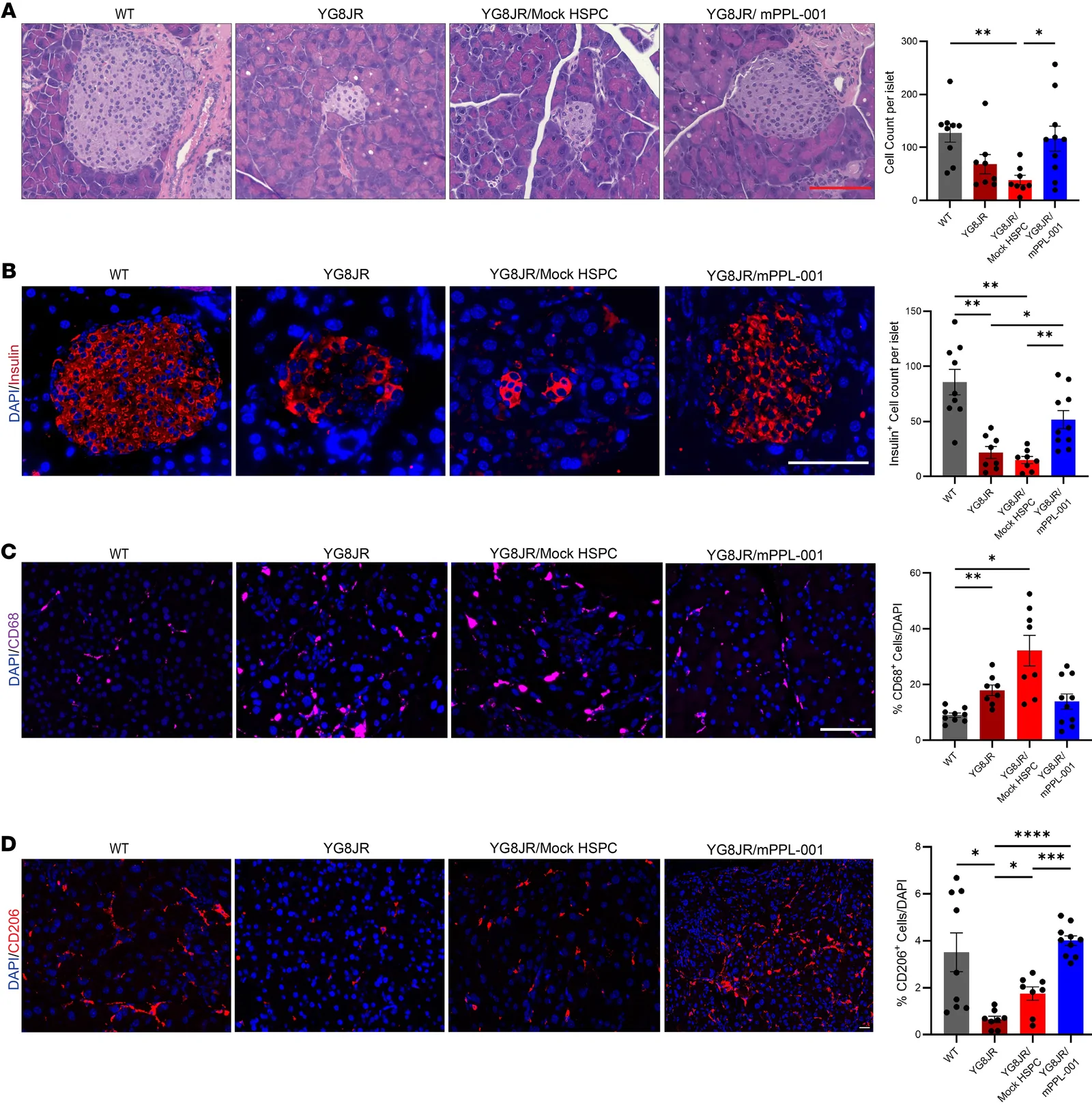
mPPL-001 transplantation preserves insulin-producing β cells of pancreatic islet of Langerhans by attenuating inflammation. (**A**) Representative H&E images and quantification of islet of Langerhans’ cellularity in pancreas (WT = 6M/3F, YG8JR = 4M/4F, mock = 5M/3F, and mPPL-001 = 9M/1F). 3–7 islets per mouse were quantified (**B**) Representative immunofluorescence images and quantification of insulin-producing β cells. 3–7 islets per mouse were quantified. (**C**) Representative immunofluorescence images and quantification of pancreatic inflammatory CD68^+^ macrophages. (**D**) Representative immunofluorescence images and quantification of pancreatic antiinflammatory CD206^+^ cells. Scale bars: 50 μm. Data are presented as mean ± SEM, analyzed by 1-way ANOVA. *P ≤ 0.05, **P ≤ 0.01, ***P ≤ 0.001, and ****P ≤ 0.0001.

## Version 1 10/15/2026

Electronic publication
