# Supplementary material for: Edited HSPCs rescue frataxin deficiency and inflammation-driven multisystemic pathology in Friedreich’s ataxia mice
Source: J Clin Invest. 2026 Oct 15;136(20):e209546. doi: 10.1172/JCI209546 (PMC13668640; doi:10.1172/JCI209546)
Supplement: Supplementary file 1 [file jci-136-209546-s161.pdf]

Edited HSPCs rescue frataxin deficiency and inflammation-driven neurologic, cardiac and pancreatic pathology in Friedreich's ataxia

Anusha Sivakumar[1], Alexis N. Corl[1], Keneth Jordan Muñoz[1], Subhashini Arumugam[1], Betul B. Gunes[1], Angelyn B. Solis[1], Rishika Murthy[1], Lilas Rony El-Hachim[1], Donald P. Pizzo[2], Ian A. Blair[3,4], Teerapat Rojsajjakul[3,4], Clementina Mesaros[3,4], Xinlong Wan[1], Joseph N. Rainaldi[1], Rafael A. Badell Grau[1], Veenita Khare[1], Kevin Eric Thai[1], Kavya Biederman[1], Stephanie Cherqui[1*]

[1] Department of Pediatrics, Division of Genetics, University of California, San Diego, La Jolla, CA 92093, USA
[2] Department of Pathology, University of California, San Diego, La Jolla, CA, USA
[3] Penn/CHOP Friedreich's Ataxia Center of Excellence, Philadelphia, PA 19104, United States.
[4] Center of Excellence in Environmental Toxicology, Department of Systems Pharmacology and Translational Therapeutics, Perelman School of Medicine, University of Pennsylvania Philadelphia, PA 19104, United States.

Guide to Supplementary Materials

Supplementary Figure 1 provides supporting characterization of mPPL-001 to assess excision specificity of the sgRNAs (UP4 and DN4) and preservation of HSPC reconstitution potential, *in vitro.*

Supplementary Figure 2 demonstrates relationship between engraftment of mPPL-001-dereived cells and neurological functional outcomes.

Supplementary Figure 3 identifies GAA excision threshold in mPPL-001 necessary for consistent therapeutic benefits.

Supplementary Figure 4 provides supporting transcriptomic analysis that demonstrate rescue of disease-associated transcriptional programs in mPPL-001 recipients

Supplementary Figure 5 presents baseline cardiac measurements that support interpretation of the treatment response in mPPL-001 recipients

Supplementary Figure 6 contextualizes effects of mPPL-001 on the pancreatic phenotype in FRDA

Supplementary Tables 1 and 2 provide the gene-level results supporting the transcriptional rescue in mPPL-001 recipients.

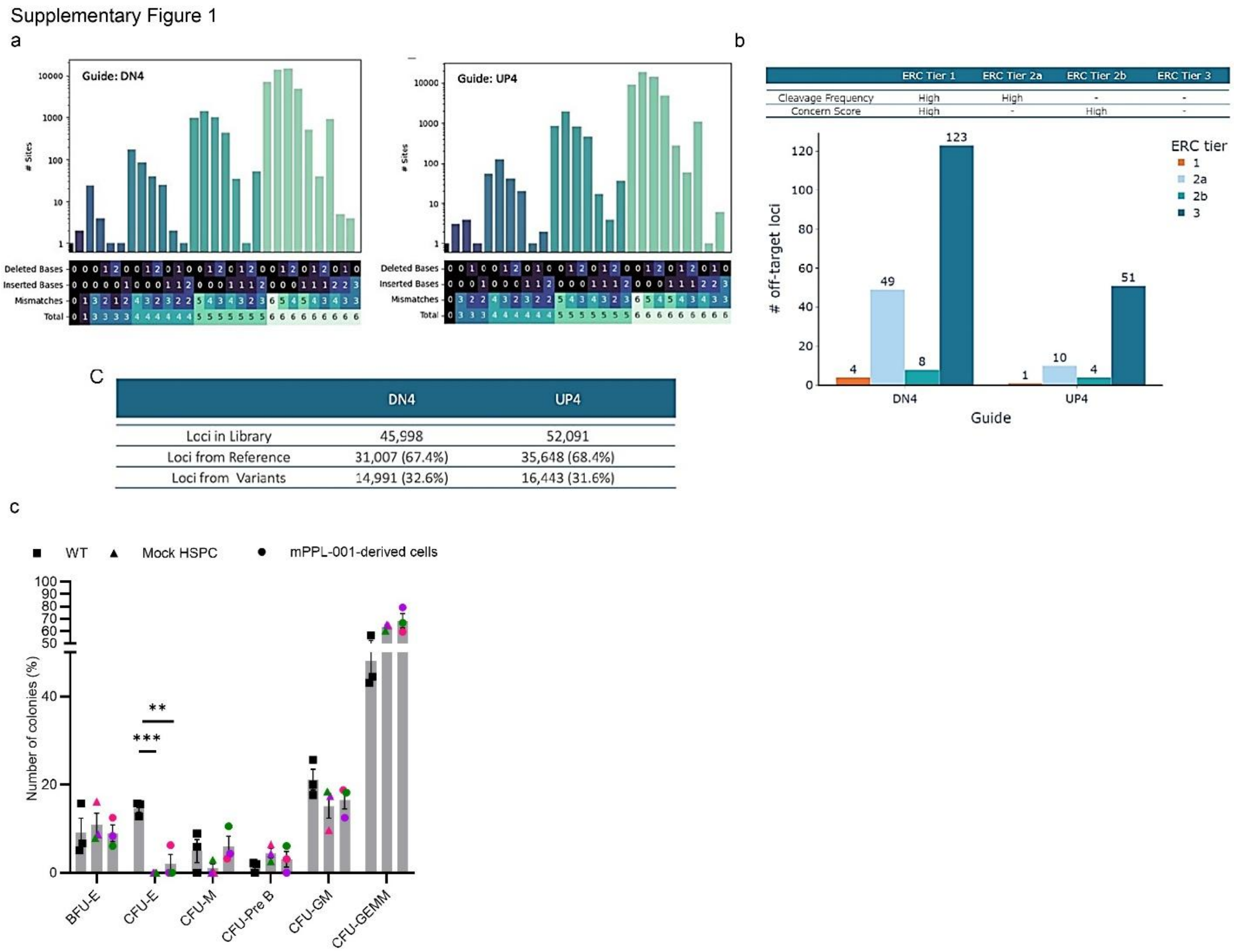

Supplementary Figure 1. Characterization of mPPL-001 editing and hematopoietic reconstitution. (a) ONE-seq nomination profiles for DN4 and UP4 sgRNAs. **(b)** *In vitro* cleavage assay nomination of putative off-target sites for UP4 and DN4. **(c)** Quantification of *in vitro* differentiation potential of HSPCs using Colony forming units (CFU) assay (BFU-E: Burst Forming Unit-Erythroid; CFU-E: Colony Forming Unit-Erythroid; CFU-M: Colony Forming Unit-Macrophage, CFU-Pre B: Colony Forming Unit-Pre-B cell, CFU-GM: Colony Forming Unit- Granulocyte, Macrophage; CFU-GEMM: Colony Forming Unit-Granulocyte, Erythroid, Macrophage, Megakaryocyte).

Supplementary Figure 2

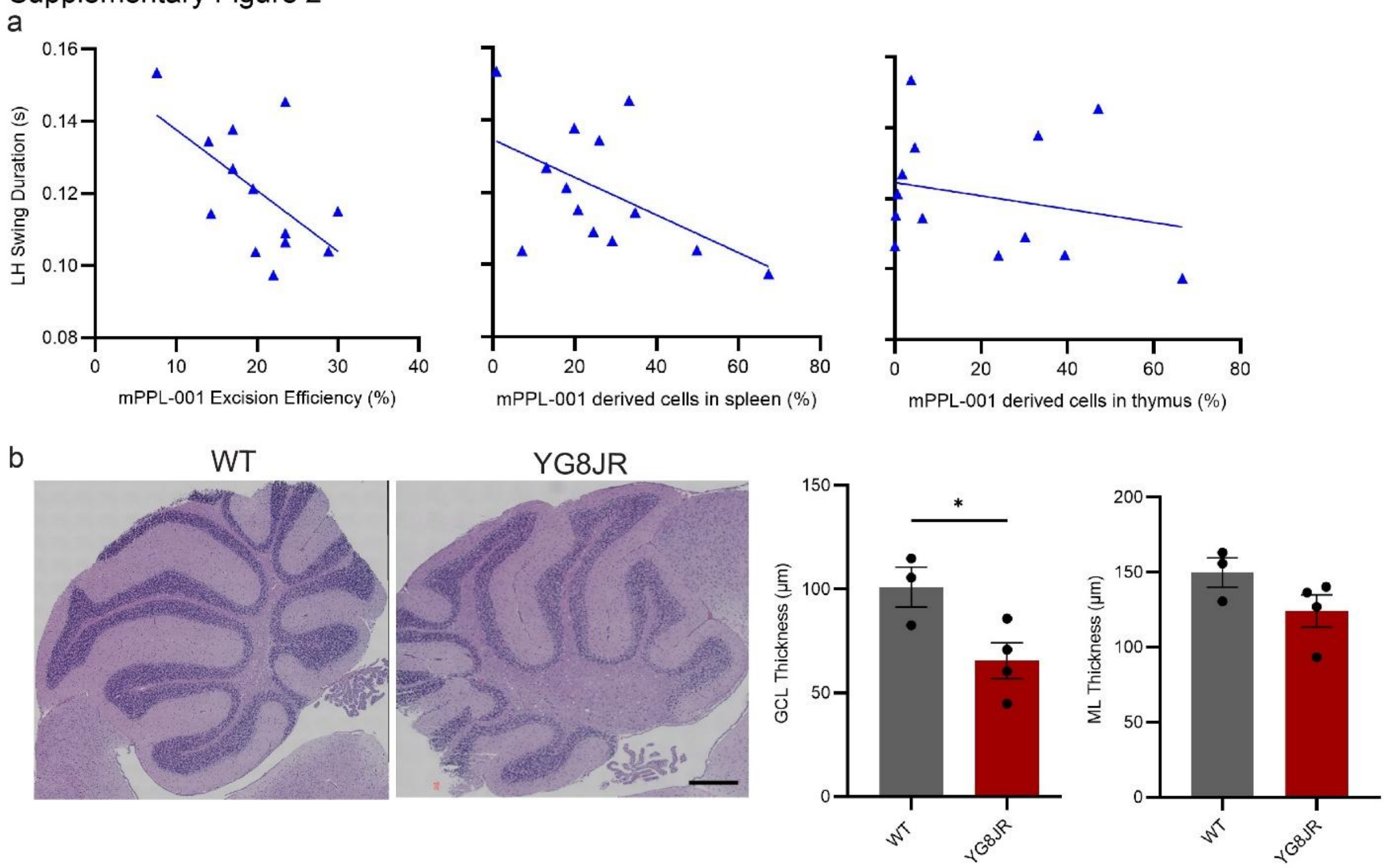

Supplementary Fig 2. Correlation analysis of mPPL-001 transplantation with gait improvements and cerebellar pathology in YG8JR mice. (a) Regression analysis of Left Hind (LH) swing duration *vs*. mPPL-001 excision efficiency in the input product (p=0.03; F=5.94; r^2=0.35) and mPPL-001-derived cells in spleen (p=0.06; F=4.097; r^2=0.27) and thymus (p=0.4; F=0.6; r^2=0.05. **(b)** Representative H&E-stained cerebellar sections and quantification of granular cell layer (GCL) and molecular layer (ML) thickness in 2.0-2.5 months old WT and YG8JR mice (age of transplant). Scale bar = 100µm. Data are presented as Mean ±SEM and analyzed by unpaired *t*-test. Significance is set at *p ≤ 0.05, **p ≤ 0.01, ***p ≤ 0.001 and ****p ≤ 0.0001.

Supplementary Figure 3

a

Groups	h*FXN* GAA Excision Efficiency in mPPL-001
Group 1	19.80
Group 2	14.00
Group 3	7.60
Group 4	14.30
Group 5	28.80
Group 6	17.00
Group 7	22.00
Group 8	30.50
Group 9	23.50
Group 10	28.30

b

mPPL-001 Excision Efficiency (%)	mPPL-001 recipients	Bone marrow chimerism (Mean)	Cerebellar FXN (Change over Mock HSPC)	Left hind swing duration (Shift Towards WT)	Coupling LH-RH (Shift Towards WT levels)
7.60	n=1	0.28	Similar to Mock	Similar to Mock	Similar to Mock
14.00	n=1	0.90	Near WT levels	Similar to Mock	Partial shift towards WT, improved relative to Mock
14.30	n=1	7.30	Near WT levels	Near WT levels	Similar to Mock
17.00	n=2	3.29	Near WT levels	Near WT levels	Near WT levels
19.80	n=1	1.90	Near WT levels	Near WT levels	Near WT levels
22.00	n=1	9.00	Near WT levels	Near WT levels	Near WT levels
23.00	n=3	13.43	Near WT levels	Partial shift towards WT, improved relative to Mock	Partial shift towards WT, improved relative to Mock
28.80	n=1	36.10	Near WT levels	Partial shift towards WT, improved relative to Mock	Near WT levels
28.30	n=1	2.57	Near WT levels	Near WT levels	Near WT levels
30.50	n=1	3.49	Near WT levels	Near WT levels	Near WT levels

Color code
Similar to Mock
Partial shift towards WT, improved relative to Mock
Near WT levels

Supplementary Figure 3. Cohort-level relationship between mPPL-001 GAA excision efficiencies and therapeutic response. (a) h*FXN* GAA excision efficiencies in input mPPL-001 across treated cohorts. **(b)** Summary of cohort-level therapeutic responses in relation to mPPL-001 excision efficiency.

Supplementary Figure 4

a

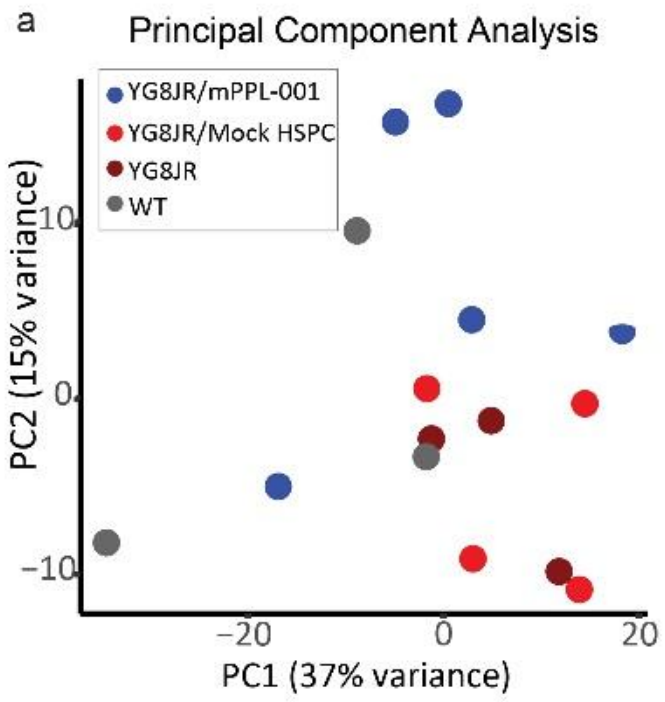

b

Permutational Multivariate of Analysis Variance

PERMANOVA Global					
	Df	SumOfSqs	R2	F	Pr(>F)
Model	3	12887.736	0.322	1.740	0.049
Residual	11	27152.890	0.678	NA	NA
Total	14	40040.626	1	NA	NA

c

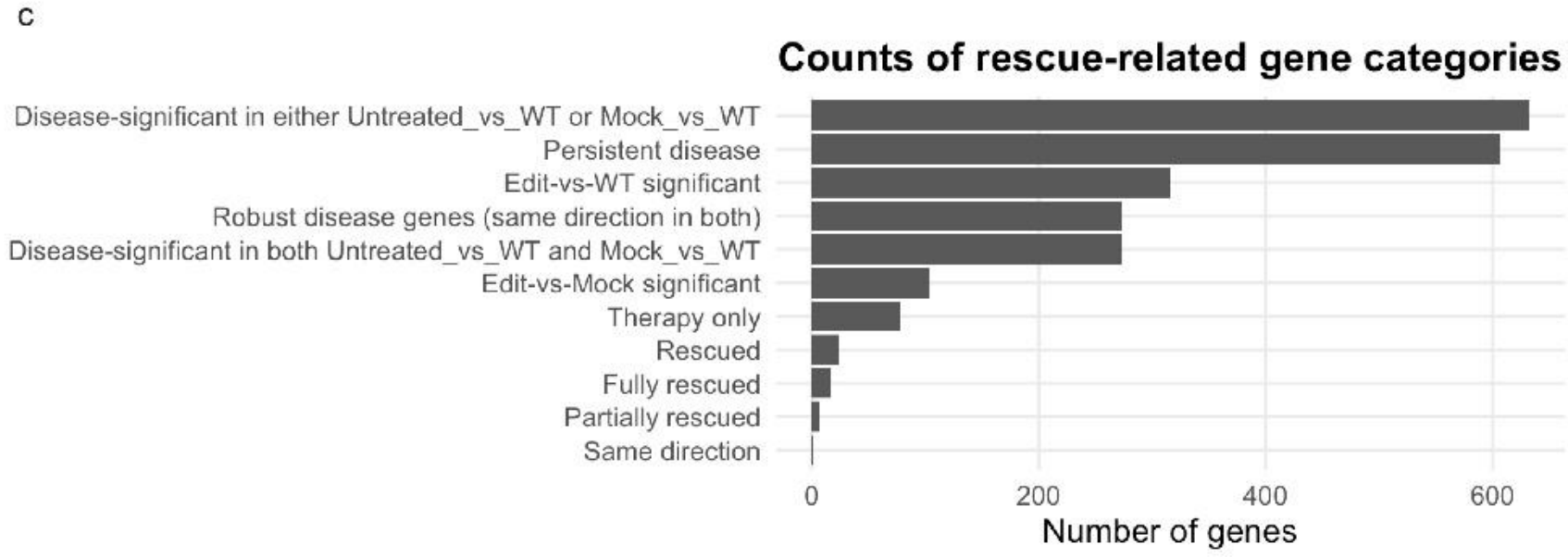

d

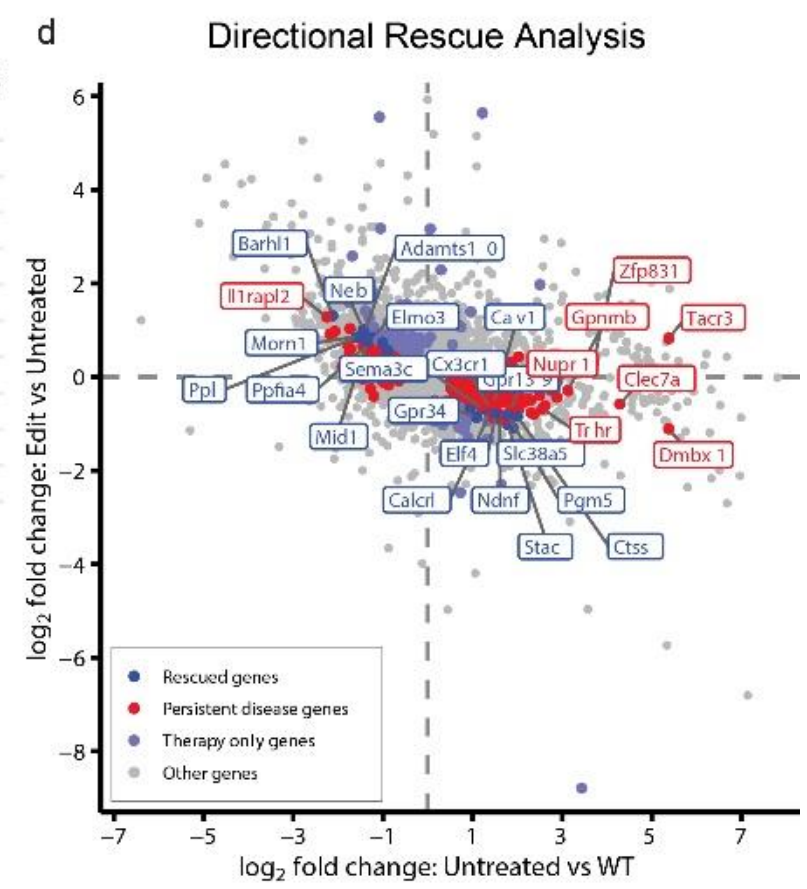

Supplementary Figure 4. mPPL-001 induces partial rescue of disease-associated cerebellar transcriptional changes in YG8JR mice. (a) Principal component analysis of cerebellar transcriptomic profile. **(b)** Global PERMANOVA of cerebellar transcriptomic profiles. **(c)** Counts of rescue-related gene categories. **(d)** Directional rescue analysis comparing disease-associated transcriptional changes (untreated *vs.* WT) with mPPL-001 associated changes (mPPL-001 *vs.* untreated).

Supplementary Figure 5

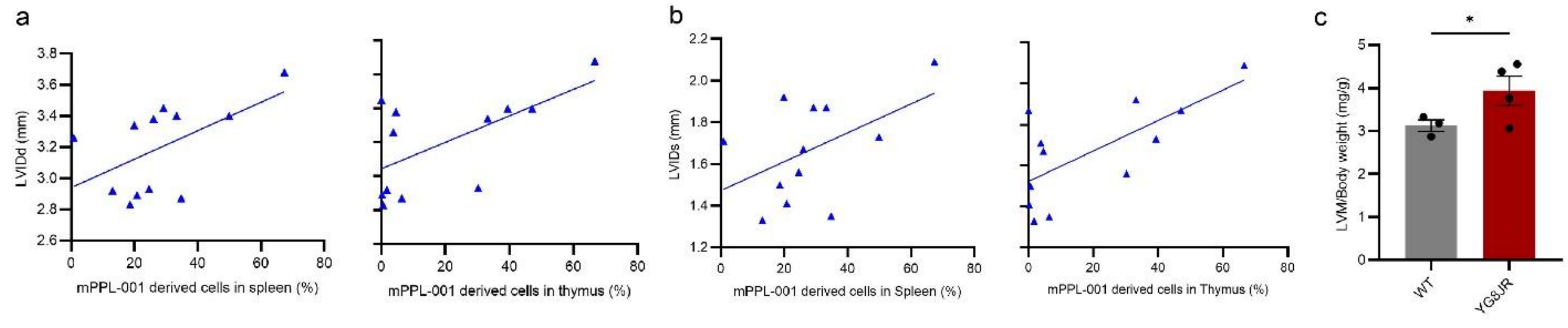

Supplementary Figure 5. Echocardiographic parameters correlate with hematopoietic engraftment of mPPL-001-derived cells. (a) Correlation of LVIDd *vs.* mPPL-001-derived cells engrafted in spleen (p=0.06; F=4.258; r^2=0.29) and thymus (p=0.03; F=0.616; r^2=0.38). **(b)** Correlation of LVIDs *vs.* mPPL-001-derived cells engrafted in spleen (p=0.1; F=3.2; r^2=0.24) and thymus (p=0.01; F=9.511; r^2=0.48). **(c)** M-mode echocardiography measurement of left ventricular dimensions in 2.0-2.5 month old YG8JR (age of transplant) and age-matched WT mice. Data are presented as Mean ±SEM and analyzed by unpaired *t*-test. Significance is set at *p ≤ 0.05, , **p ≤ 0.01, ***p ≤ 0.001 and ****p ≤ 0.0001.

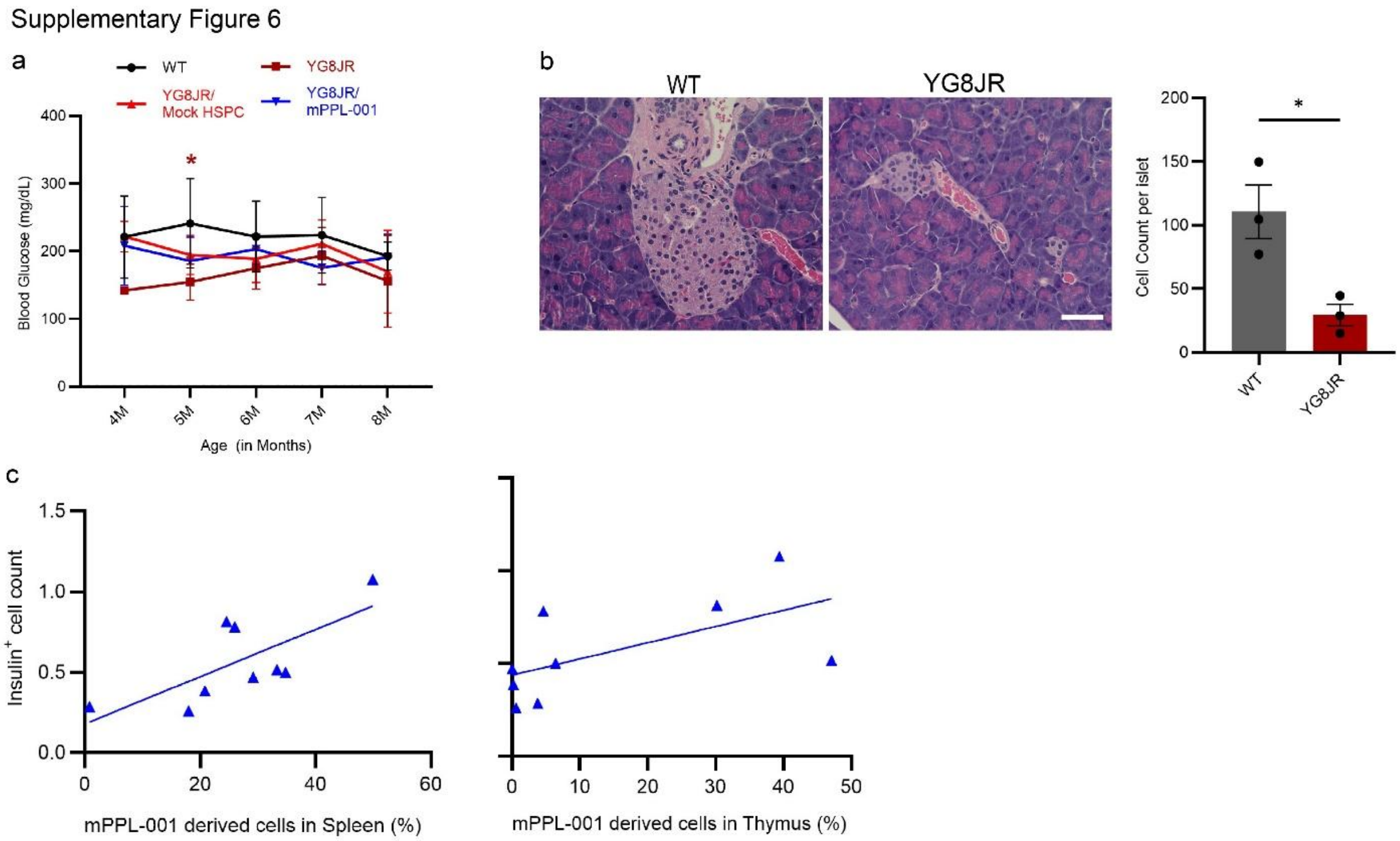

Supplementary Figure 6. Abundance of Insulin positive β-cells in pancreas correlate with hematopoietic engraftment of mPPL-001-derived cells. (a) Periodic fasting body glucose measurement of mice of all cohorts up to 8 months of age. * Represents significance at 5 months between WT and untreated YG8JR mice. **(b)** Representative images of H&E-stained pancreatic sections and quantification of cell count within islets in 2.0-2.5-month-old YG8JR mice compared to age-matched WT controls (WT=3, YG8JR=3). 3-5 islets per mouse were quantified. Scale bars=50μm. **(c)** Regression analysis of Insulin$^+$ β-cell numbers *vs*. engraftment of mPPL-001-derived cells in spleen ($p=0.02$; $F=7.66$; $r^2=0.52$) and thymus ($p=0.08$; $F=4.023$; $r^2=0.36$).
